# The effects of occupational and mental stress among home care rehabilitation professionals working during the COVID-19 pandemic: An exploratory qualitative study

**DOI:** 10.1177/10848223231225246

**Published:** 2024-01-30

**Authors:** Aaron S. Howe, Kevon Jules, Jeremy KCD Tan, Raabia Khan, Anson KC Li, Brydne Edwards, Emily C King, Sonia Nizzer, Basem Gohar, Amin Yazdani, Ali- Bani-Fatemi, Vijay Kumar Chattu, Lindsay Sinclair, Mhairi Kay, Behdin Nowrouzi-Kia

**Affiliations:** 1 Restore Lab, Department of Occupational Science and Occupational Therapy, Temerty Faculty of Medicine, University of Toronto, Toronto, Ontario, Canada; 2 Department of Occupational Science and Occupational Therapy, Temerty Faculty of Medicine, University of Toronto, Toronto, Ontario, Canada; 3 VHA Home HealthCare, Toronto, Ontario, Canada; 4 Dalla Lana School of Public Health, University of Toronto, Toronto, Ontario, Canada; 5 KITE - Toronto Rehabilitation Institute, University Health Network, Toronto, Ontario, Canada; 6 Department of Population Medicine, University of Guelph, Guelph, Ontario, Canada; 7 Centre for Research in Occupational Safety and Health, Laurentian University, Sudbury, Ontario, Canada; 8 Canadian Institute for Safety, Wellness & Performance, Conestoga College Conestoga College Institute of Technology & Advanced Learning, Kitchener, Ontario Canada; 9 Center for Global Health Research, Saveetha Medical College and Hospital, Saveetha Institute of Medical and Technical Sciences, Saveetha University, Chennai, India; 10 Center for Evidence-based Strategies, Global Health Research and Innovations Canada (GHRIC), Toronto, Canada; 11 Centre for Addiction and Mental Health, Toronto, Ontario, Canada; 12 Professional Support Services, Toronto District School Board, Toronto, Ontario, Canada; 13 Toronto Western Hospital, University Health Network, Toronto, Ontario, Canada

**Keywords:** Homecare, occupational therapy, rehabilitation, quality of life, work-related stress, COVID-19 pandemic, healthcare, Ontario, Canada

## Abstract

Home care rehabilitation professionals (hcRPs) provide health services for clients with a broad range of medical conditions. During the COVID-19 pandemic, home care rehabilitation professionals experienced exacerbations of pre-existing work-related stressors, increased risk of transmission of the COVID-19 virus, reduced resource availability, greater workloads, and staffing shortages. The primary aim of this study was to examine the experience and impact of occupational and mental stress on hcRPs working during the COVID-19 pandemic. Semi-structured interviews were conducted with 24 hcRPs working in Ontario, Canada during the COVID-19 pandemic. Inductive thematic analysis was used to interpret and organize the data into conceptualized themes. Interview data was organized into three themes: (a) unique challenges of a home care rehabilitation professional, (b) COVID-19 exacerbations of home care occupational and mental stress, and (c) personal and workplace coping strategies. Many participants reported reducing their hours or taking on adjunctive roles in different clinical settings outside of home care due to work-related stress exacerbated by the COVID-19 pandemic. With a focus on the effects of COVID-19 on the practice of home care, this study provides a unique perspective on the challenges experienced by hcRPs during an emergent and evolving global public health concern. The exploratory nature of this research works towards providing a framework of factors to be addressed when creating sustainable healthcare interventions, as well as recommendations to support hcRPs to benefit both the community and health-care providers.

## Introduction

Home care plays an essential role in decreasing mortality and hospitalizations, as well as reducing overall healthcare costs.^1^ As an important part of the home care system, in-home rehabilitation professionals provide health services and care for clients with a broad range of medical conditions.^2^ Their clinical services are delivered at a client’s home, including in residential care facilities (e.g., retirement homes). This form of rehabilitation emphasizes independent living while addressing and improving clients' quality of life and health status.^3^ Home care rehabilitation professionals (hcRPs) provide rehabilitation services and adaptive strategies (e.g., mobility and client’s safety) to clients of all ages with a variety of medical diagnoses, including but not limited to cerebral palsy, developmental delay, arthritis, chronic obstructive pulmonary disease, dementia, and cancer.^4,5^ Home care clients may receive treatment from various rehabilitation specialists including occupational therapists, physiotherapists, occupational and physical therapy assistants, social workers, speech-language pathologists, and dieticians.^5^ Approximately 6% of Canadian households reported using home care services in 2021, with older adults and individuals in low socioeconomic suburban neighborhoods representing the largest proportion of users.^6^

Although occupational stress and burnout can be present in all sectors of healthcare, hcRPs may experience unique and specific challenges within their occupation compared to other clinical settings.^7^ Within intimate client spaces, hcRPs must adapt to working in home environments that are not designed as care environments and accommodate person-specific factors to appropriately fulfil their clinical duties.^8^ While the primary focus remains on improving independence in the home and supporting daily functioning (e.g. toileting, dressing, transferring, basic mobility, and communication), these considerations alter the experience and provision of services for both the clients and the rehabilitation professional.^9^ In addition to challenges encountered by their facility-based colleagues such as overcoming communication barriers, psychoeducation, and assistive device training, hcRPs provide care in a context of greater environmental adaptation with increased social and caregiver involvement in the client’s care and struggles with provider isolation.^9,10^

Recent qualitative studies of healthcare professionals have identified that self-reported occupational burnout was associated with sentiments of moral distress regarding the health status of their clients, inflexible schedules, lack of role appreciation, interpersonal difficulties with clients and their families, changes to workflow, adaption to new technologies, shortages of workplace resources and staff, and lack of time to complete daily work tasks.^11-14^ These factors have also been found in samples of rehabilitation providers, including occupational therapists,^15-17^ physiotherapists,^18^ and speech therapists.^19^ It has also been characterized that positive perceived working conditions and professional identity have been negatively correlated with burnout.^20-22^ A literature review^23^ examining job satisfaction and well-being in speech-language pathologists identified workload/caseload size, professional support, and salary as factors associated with wellbeing. Another study^24^ examined resiliency factors in a sample of occupational therapists and found that negligible stress symptoms in therapists were associated with experiencing a low workload, high control, and a greater sense of community and justice. To our knowledge, there are no studies examining the impact of these factors on healthcare professionals that provide home care services.

The COVID-19 pandemic exacerbated challenges throughout Canada’s vulnerable healthcare system, including home care. Healthcare providers encountered increased risk of exposure to the COVID-19 virus, reduced workplace resources availability, greater workloads, and staffing shortages.^6,17^ As a result, healthcare workers with no previous intentions to retire reported interest in leaving their job or changing occupations.^25^ It was reported that 63.2% of healthcare workers listed occupational stress and burnout as the most common reasons for occupational change.^17^ Occupational stress encompasses the physical and emotional harm arising from excessive work-related demands that exceed the abilities of the worker or provided resources.^26^ Prevalent sources of occupational stress may include a lack of reward or control, reduced workplace resources, a sense of threat, social isolation, and insufficient social support.^27^ As a result of prolonged and poorly managed occupational stress, burnout can emerge in the form of physical and emotional exhaustion. Occupational burnout can be defined by the feeling of energy depletion or exhaustion, increased mental distance from one’s job, and reduced professional efficacy.^28^

Currently, the effects of COVID-19 on hcRPs in Canada are unknown, especially in the context of occupational and mental stress. Using an inductive qualitative approach, this study explored the experience and impact of occupational and mental stress on hcRPs working during the COVID-19 pandemic. Upon examination of these experiences, recommendations were developed to support hcRPs through future public health crises.

## Methods

### Sample Recruitment and Setting

Participants were recruited during the COVID-19 pandemic using voluntary convenience sampling through invitations emailed to all rehabilitation providers at a large home care organization in Ontario, Canada. This organization provides 24/7 home and community care services to people of all ages and diverse cultural backgrounds. All participating rehabilitation professionals worked in home and community care, which could include providing services in a client’s home, school, a long-term care facility, or a retirement home. Participant inclusion criteria were a) adult (18-65 years old); b) an ability to speak and write in English; and c) working as a regulated hcRP in Ontario (e.g., physiotherapist, occupational therapist, speech-language pathologist, dietician, social worker) during the COVID-19 pandemic (March 2020 onwards).

Thirty-two hcRPs who expressed interest in the study were contacted by research staff at the University of Toronto through email to invite them to participate in a semi-structured interview and share their occupational experiences during the COVID-19 pandemic. All participants provided written informed consent prior to enrollment in the study. Eight of these hcRPs did not respond to this email communication; the remaining 24 formed the study sample. A $50 gift card honorarium was provided to all participants upon interview completion. Research ethics approval was received from the University of Toronto’s Research Ethics Board (RIS Protocol #42041).

### Interview Procedures

Semi-structured interviews were approximately 60 minutes long and conducted on Microsoft Teams^®^ software (Microsoft, 2022) by a trained Master’s level research analyst (ASH). This researcher was male and had previous experience in conducting semi-structured interviews, focus groups, and facilitating psychological group therapy. Rapport was established with each participant. The research training and background of the interviewer, and the interest and goals of the research were communicated. Confidentiality and consent were discussed with each participant prior to the commencement of the interview. Demographic data was collected at the beginning of the interview to provide context to the qualitative findings. Employee assistance resources were made available to each participant.

The interviews were digitally video- and audio-recorded through Microsoft Teams (version 1.5) and transcribed verbatim through an external professional service. Previous qualitative studies have suggested a sample size of at least 12 interviews to achieve data saturation.^29,30^ Initially, we proposed to complete 12 interviews, however data saturation was not achieved based on the scope of study and pragmatic considerations (e.g., participant response rate, emergent new themes, and resource availability). The sample size was subsequently increased until additional interview data no longer contributed to thematic development. Interviews were completed during two time periods that were two years after the inception of the COVID-19 pandemic: May 25 – June 17, 2022, and November 3 – 18, 2022, due to participant availability. Repeat interviews were not conducted.

The interview guide was developed in collaboration with the partnering organization and from review of the existing literature on burnout and occupational stress in healthcare workers.^31,32^ The questions posed were constructed to capture aspects of occupational stress and burnout, such as emotional exhaustion and depersonalization. Questions within the interview guide were as follows: “Tell me what is it like work during the COVID-19 pandemic; Tell me about your preferred area of work at your organization; Describe a typical work day for me; What are some of the types of stress in your work setting; Do you feel these stressors are specific to your employment or industry-wide; Did you feel supported by your employer in dealing with these stressors; Tell me about your quality of work life living in the homecare sector; If you could change something to improve your quality of work life, what would it be; How has working in the homecare sector affected your mental health; Has your perception and/or attitude changed about your work in the past two (2) years; Have you considered a career change during the COVID-19 pandemic and if so, what other careers have you considered; Has this influenced your work-life balance and life enjoyment; Have you found ways to manage the stresses you have experienced while working during the COVID-19 pandemic.”

### Analysis

Socio-demographic data was summarized and expressed as frequency and percentages for the following variables: sample size, sex, rehabilitation profession, change in home care employment since start of pandemic, and current area of practice. Years of service and age variables were expressed as means and standard deviation. Descriptive statistics were calculated using IBM^®^ SPSS^®^ version 27.

The transcribed interviews were analyzed using Braun and Clarke’s six-stage inductive thematic analysis.^33^ Stage(s) 1 and 2: Each transcript was reviewed to enable data familiarity, and to ensure a comparable dataset for collaborative review within the coding team. Any inconsistencies (e.g., long pauses, incorrect non-verbal communication or gestures, incorrect spelling of clinical terms, and incorrect verbal expressions) in the data was resolved by reviewing the interview audio-recording and comparing it to the transcribed material and observational interview notes simultaneously. Transcripts were not returned to participants for comment or corrections, however observational notes were taken after interview completion to maintain rapport and for data accuracy when reviewing the transcripts. Familiarization with the data was a recursive process and the interview transcripts were reviewed multiple times by each member of the analysis team independently prior to coding the data. Each team member kept their own reflexive journal of their impressions of the data.

Stage 3: Initial impressions and notes of the data were collected to spur further discussion in weekly research team meetings. Next, the research team performed open coding of the data independently for the first 12 interview transcripts. Coding was performed by the first 5 authors who were from different academic and research backgrounds (e.g. medicine, psychology, kinesiology, occupational therapy). Open coding was completed by highlighting specific data sections as nodes and annotating notes within the transcript. Then, a coding meeting was organized to create a codebook to be used to code the remaining transcripts after a detailed description of each consensus code was generated. Debriefing meetings were held to discuss codes that arose after review of the 12 interview transcripts to ensure no codes were missing for thematic development. It was determined after these debriefing meetings that further interviews were required to fully develop emerging themes from the data that were not fully support by prior participant experiences. Stage(s) 4 and 5: Once data saturation was achieved, the remaining transcripts were coded, and discussions were held to organize and collate the data into fully conceptualized themes. The themes were refined with clear explanations through weekly research meetings with the analytical team. The data was interpreted inductively to ensure personal accounts and experiences of the hcRPs were exclusively guiding conceptual theme development.^29^ Stage 6: Evidence-based recommendations were developed through a data-based approach of considering the lived experiences of the hcRPs though each stage of thematic development. Recommendations were refined through recursive analysis of the data and consultation with members of the research team practising occupational therapy and/or familiar with professional home care experiences. Thereafter, the existing literature was consulted to examine the evidence that supported these developed recommendations for health care professionals working in similar healthcare settings. The qualitative analysis was conducted using NVivo 12 software.^34^

### Trustworthiness and Rigor

Methodological rigor was achieved through collaborative efforts of the first five authors to assure researcher triangulation through coding consistency and interview data obtained from multiple sources.^30,33^ These team members were from multiple disciplines including Psychology, Occupational Therapy, Kinesiology, and Medicine with different levels of research and clinical experience. Each coder kept their own reflexive journal to contribute their own interpretations of the data during familiarization with the transcripts and during each stage of coding. The remaining authors and corresponding author were involved in the latter stages of analysis, during theme refinement and write-up. This supported further researcher triangulation, as the additional authors were familiar with the home care sector, worked as occupational therapists, and/or have worked as hcRPs with the partner organization themselves. Reflexive journaling and audit trails of coding and thematic development were archived to support trustworthiness of the data. The first author kept a summarized reflexive journal electronically and was responsible for updating the consensus decision-making after each weekly research meeting. Observational interview notes regarding personal feelings, biases, and insights immediately after the interview were also stored in the reflexive journal. Systematic communication between all members of the coding team ensured audit trails and mutually agreed upon concepts were monitored in weekly research meetings through comparison of each member’s reflexive journal.

## Results

Twenty-four female hcRPs were interviewed. Most were working as occupational therapists (n=15), and the remaining sample was composed of physiotherapists (n=5), speech-language pathologists (n=2), a dietician (n=1), and a social worker (n=1). Half of the participants reported reducing their hours or taking on adjunctive roles in different clinical settings (e.g., hospital, clinic-based) outside of home care due to work-related stress exacerbated by the COVID-19 pandemic. One of the participants was in the process of transitioning out of home care into a non-clinical support role. Many participating hcRPs worked as self-employed contractors and worked for more than one employer during the COVID-19 pandemic. A summary of the sociodemographic data is provided in Table 1.

**Table 1.** Sociodemographic Data.

	Frequency (%)
Sample size	n = 24
Age	*M* = 41.2, SD = 11.0
Sex	24 F (100.0%)
Rehabilitation profession	
Occupational therapist	15 (62.5%)
Physiotherapist	5 (20.8%)
Speech language pathologist	2 (8.3%)
Dietician	1 (4.2%)
Social worker	1 (4.2 %)
Years of service	*M* = 16.2, SD = 12.0 (range: 2-40 y)
Current area of practice	
Pediatric	3 (12.5%)
Adult	9 (37.5%)
Geriatric	12 (50.0%)
Change in home care employment since start of pandemic	
No change	12 (50.0%)
Reduction in hours	11 (45.8%)
Transitioned out of home care	1 (4.2%)

Interview data were organized into three conceptual themes derived from an inductive thematic analysis approach. Three themes were identified: (a) unique challenges of a home care rehabilitation professional, (b) COVID-19 exacerbations of home care occupational and mental stress, and (c) personal and workplace coping strategies.

### Theme 1: Unique Challenges of a Home Care Rehabilitation Professional

Participants shared the challenges of working in home care compared to other, more structured clinical settings, such as hospitals, prior to the COVID-19 pandemic. In home care, participants reported that they experience occupational stress and fatigue from planning their daily commutes and preparing for the visits to the homes of multiple clients. They also mentioned that they must take additional time for travel to their office for procurement of supplies for visits. They emphasized the difficulties of navigating unpredictable, and sometimes unsafe, care environments as they visit client’s homes. Home environments with unsanitary or unsafe conditions (e.g., drug usage, bed bug infestations, safety hazards) have resulted in mental stress for some participants.

*P17: “I saw a palliative client and he fell during my visit. I couldn’t get him up. I had to wait close to an hour. Three firemen showed up and we got him into the bed. That was incredibly stressful. But stuff like that happens all the time. The next day I remember going in this woman’s apartment and sitting down and then the woman reveals that she has bedbugs and that’s why she does not use [her] bed. Here you are, you are already sitting down, which normally I wouldn’t do if I know there is bedbugs in the unit, but it is too late. There is all kind of stresses. I can go on and on about homecare.”*

*P2: “You deal with traffic, you deal with, you know, uncertain things, right. You could be driving 15, 30 minutes to the client. And they confirmed yesterday you're going to see them at two o'clock, you drive there, and they're not there. And then you have to call around and ask, and you have to be like, OK, where did you go? And then sometimes it'd be like, oh, you know, I just went shopping, or, oh, I had this appointment, but I totally forgot to tell you. So that's, those were kind of some of the stressors I guess, if I, you know, get no showed. . . I guess parking, sometimes really difficult to find parking. I mean, when you go out to see people, you deal with the weather, right. Sometimes in the winter, you really got to look for the weather forecast and, and plan accordingly. You don't want to be driving out when it's about to snowstorm. Yeah, and then I guess, just in general, you don't know what you're expecting when you enter someone's home?”*

Study participants reported that there are unique client-provider expectations that are specific to home care. Some participants spoke about feeling devalued when entering client’s homes as they felt they were disrespected by clients more than colleagues working in hospital settings. In some instances, clients did not understand that their home is a workplace for the hcRPs and therefore did not appreciate that it must be an occupationally safe working environment during these care visits.

*P15: “Going into client’s homes, it is kind of challenging sometimes too. There is that power dynamic where you are going into the client’s home but you are providing a service. They almost feel like they can be disrespectful towards you.”*

There can be a particularly broad range of client issues that require attention during home care visits, and the extent of these can amplify the workload and stress of hcRPs. Many of the participants worked with older adult clients and stated that often these individuals are medically complex and have many caregivers supports. In working with these clients, the home care visits are often prolonged and require extra effort related to mobility, communication, and administrative tasks. Recognition of the greater needs of these clients can also result in greater mental stress and guilt for the hcRP that can lead to accepting cases beyond their preferred or expected workload. These existing occupational stressors were identified as impactful to the participants, particularly as the stressors are managed independently by hcRPs as they work in the client’s home alone and without immediate access to or assistance from other healthcare workers.

*P14: “I've noticed that I have a certain capacity working with geriatric patients. Because it's tiring, it's more tiring than working with adults, or younger people. Because they need certain mental engagement and physically, they are very fragile, and they are usually medically very complex. Cognitively, usually impaired, dementia, like memory deficits, hearing problems, vision deficits. So, there is always on top of that family expectation, you must deal with so many people. Besides, you're taking care of your patient.”*

### Theme 2: COVID-19 Exacerbations of Home Care Occupational and Mental Stress

Most of the hcRPs interviewed in this study reported experiencing increased occupational and mental stress in the context of the COVID-19 pandemic. A few participants subjectively reported experiencing work-related burnout. Several factors were reported as exacerbating work-related stress in this sample of hcRPs, including heightened challenges in the workplace environment and a lack of healthcare resources, reduced colleague collaboration and socialization, and feeling unable to meet pre-pandemic levels of referrals.

### Subtheme 1: Workplace environment and reduced healthcare resources

Early on during the COVID-19 pandemic, many of the hcRPs reported that they had difficulties adapting their clinical services to the evolving health and safety guidelines. Additional paperwork and screening protocols to follow before clinical visits with clients created additional administrative work. Some had struggled to manage with system-wide shortages of personal protective equipment (PPE); others reported inconsistencies in training and policies between different employers. They discussed their duty to regularly change or sanitize PPE and other work equipment between clients to reduce the risk of COVID-19 transmission between home visits. The fear of disease transmission, in general, was reported to contribute to significant anxiety and stress about transmitting the virus to other clients during visits to private homes, retirement homes, and to their family and friends. This anxiety also transferred to social and non-work events where they would limit their participation in social activities. Some participants reported that the PPE itself would cause anxiety and panic due to difficulty breathing or their face shields fogging when performing their clinical duties.

*P9: “It is hard to maintain it. You know it is hard to be conscientious [about infection control] all the time. It is really hard, but my feeling is that you know if I carry it [the COVID-19 virus] from one person to another that would be incredibly awful. I would feel guilty and even though I never meant to do [it].*

*P23: “Sometimes when I was wearing my N95 I was wearing my face shield, sometimes, I can't breathe. I need to get out from the retirement home, sometimes I have patients at the retirement home, I need to get out there because I can't breathe. So definitely it puts a lot of anxiety, stress, overwhelming and then knowing that sometimes you come home, and was I bringing the virus in our household? So, right away, as soon as I get into our place, I have to change and then put my clothes into the washing and – it was a lot to take in.”*

Navigating pandemic-related public health and occupational health guidelines with clients and their families added new psychosocial challenges. Often, participants reported having to educate the client about COVID-19 transmission, and that this was met with resistance and non-compliance by some clients. For example, some clients were not in-forming their hcRP about exposure or contact to someone with COVID-19, not valuing or sharing beliefs about vaccination and PPE usage, and not wearing masks either in their private home or in their retirement homes (in which they felt they should have a choice not to mask). Clients would invite additional family to the home visits (violating social distancing rules in effect at the time of the visit), and the presence of the additional family could sometimes impede clinical activities due to crowding. There were also instances where family members were dishonest about testing positive for COVID-19. Despite these challenges, the hcRPs reported that they would effortfully adapt to each situation while managing feelings of discomfort when entering a client’s home.

Many of the participants reported that their clients were increasingly medically complex and were not receiving the necessary care or services that their impairments required due to early discharge from hospitals and greater reliance on home care during the pandemic. Some participants reported increased emotional labour as some clients would share their grievances about the healthcare system; others reported providing additional referrals to clients in desperate need of personal support and community re-sources. In the context of an overwhelmed healthcare system in which access to care providers was limited, some participants stated that they would often have to address medical issues that developed because of lack of access to other health care providers, resulting in the clients having different medical needs at the time of contact with the participant than at the time of the referral. COVID-related health conditions also changed the parameters of some participants’ roles as rehabilitation professionals as they focused on mitigating long-COVID risk and planning for future care. Participating hcRPs also had to manage the stresses of supporting their clients appropriately despite limited ability to secure trial equipment, and access community and social services for their clients due to closures or suspensions of most community programs during this pandemic period.

*P24: “How your population looked was very different. Changing your role to doing more energy conservation with your COVID clients from more self-care equipment at home. Well, now you are starting to do a lot of future planning. How do we manage and mitigate the long COVID? How do we manage changes in the community in terms of referring to a personal support worker?”*

*P12: “In terms of the patients that we are seeing, that is where we see the most stress. Caregivers completely stressed out. Clients that are coming in, you know, with conditions that have gone unchecked because of lockdowns. There is a huge impact. I say it is interesting because I feel like I will always remember this time. It’s very distinct from any other time for me.”*

Ultimately, the COVID-19 pandemic had added unique complexities and variability to their workplace environment(s), reduced healthcare resource availability, and client and caregiver interactions were increasingly unpredictable and challenging.

### Subtheme 2: Lack of collaboration and socialization with colleagues

Most participants commented on a lack of collaboration with colleagues in home care during the COVID-19 pandemic due to multiple factors, including the already independent work context, the shift from in-person to virtual collaboration, and increased workloads that made it more difficult to schedule time to consult with colleagues or participate in learning opportunities. Many participants reflected that prior to the pandemic working in home care provided the benefits of autonomy and flexibility, however these participants also identified a lack of collaboration and social isolation as one of the most difficult aspects of working in home care during the COVID-19 pandemic. Some participants also felt isolated from the services and equipment vendors that they were in partnership with.

*P24: “[In home care] you feel isolated, and I can only imagine that all [home care] occupational therapists had a really hard time with this. Just all the steps involved to be able to collaborate with someone. I found that challenging.”*

*P13: “I think I felt more isolated. More isolated from my colleagues and more isolated from other services that I partner with.”*

Given the public health guidelines in Ontario, many team or clinical meetings were shifted from in-person to online during the pandemic, which for some was a barrier to work engagement and collaboration. Some of the participants stated that they believed increased opportunities for collaboration and socialization would have improved their quality of work life during the pandemic. They also referenced that opportunities for timely case discussion(s) with colleagues and case managers were limited and were more difficult to arrange due to scheduling conflicts. Additionally, the medical complexity of their clients more often required them to direct their clients to the hospital, further limiting collaboration with other hcRPs. Due to increasing referrals adding to their workload, opportunities to participate in learning or mentorship programs were limited.

Opportunities for social support were also more limited. Many relied on their friends and family as social support to discuss the challenges and stresses of their work. A few participants shared that they felt hesitant reaching out to other hcRPs for support given the fatigue and work-related stressors experienced by others. These feelings of isolation had driven most participants to consider reducing their hours of working in home care during the pandemic, allowing them to also work in a hospital or clinical-based setting to restore the collegial aspects of their professional experience.

*P8: “The supports are there, but they’re limited. I haven’t really looked into what kinds of supports [that] are there. I know that once in a while they will send out emails with a resource list of free services you can access. I have not had a chance to look into those myself but I have called them here and there. It is my own personality trait, to not want to reach out for help very much.”*

*P3: “In the community, you also don't really see your coworkers as much so you are self-directed, and you are independent. There's no one really to talk to. I would actually often bring it home to vent or talk about it to help me as an outlet.”*

### Subtheme 3: Feeling unable to meet pre-pandemic referral demands

All the hcRPs interviewed in this study discussed the difficulties of increasing workload during the pandemic. This was characterized as an increase in referrals, longer assessments and client visits, more medically complex clients, additional protocols (e.g., IPAC and screening), administrative duties for care coordination, and working outside of their clinical hours. Some of the participants experienced a greater number of referrals for clients who were neglected and lacking adequate medical attention due to the increased burden on the healthcare system during the pandemic. Therefore, they felt a personal burden to take on more clients due to the need for community home care that eventually exceeded their ability to maintain an adequate work-life balance. Some participants actively reduced their referrals during the pandemic, but found they returned to a higher caseload shortly thereafter due to financial obligations or feelings of responsibility to the clients and communities they serve. Others reported that they maintained a reduced caseload or reduced the number of hours they dedicated to home care.

For those taking on a greater number of referrals, the participants reported significant strain on their ability to perform essential aspects of their profession, such the inability to contact their new referrals in a timely manner. They also reported difficulty in establishing work-life boundaries as they often responded to work emails and completed administrative tasks after hours or on weekends to keep up with increased workloads. According to the participants, they felt that they could not effectively meet the demand of the referrals for community home care because each case was medically complex, and the rehabilitative care had to be administered in a limited number of clinical visits.

*P21: “Yeah but I think over time too given that I felt that, oh, I was an essential worker, I needed to do this to help the community and help, you know, my patients, I think I overworked, like I think I took on too much. I ended up working six days a week, seven days a week for the better part of COVID. . . There were definitely days where I felt like I was shorter with patients or shorter with my colleagues because of that burden.”*

*P10: “But sometimes my patients, they go to the hospital, and they come home soon, like too soon. Because of COVID I believe they don't keep them for long. And that affects my caseload. So, they come back, they need us really fast. In a couple of days they want us there, because they need physio, they should have been in the hospital. So that all is also really hard to manage.”*

*P20: “I think it was a month and a half that people were waiting for a phone call from me but I was just – I had too many referrals that I had picked up and that I was unable to respond to. And that was really a low point, feeling like I had failed the clients.”*

Overall, the participants felt that their clients’ expectations were greater and more complex than they had experienced when working in home care before the pandemic.

### Subtheme 4: Availability and Timeliness of Workplace Support

Workplace support was identified as an influential factor for these participants’ experiences of working during the pandemic. Some participants identified a lack of support in the context of dealing with the overwhelming referrals from the healthcare system and increased turnover rates of colleagues. In the beginning of the pandemic, there were also system-wide difficulties in financial compensation as there were different pay rates for virtual care and in-person visits. The lack of sick pay for these independent contractors was also another barrier to support. Many of the participants voiced that they felt pressure to return to in-person clinical services despite anxieties and stresses about COVID-19 transmission and reduced PPE availability. They believed this pressure was associated with a reduction in their clinical teams’ size and increased feelings of occupational and mental stress amongst their colleagues. They reported feeling unsupported by in the care environment (e.g., retirement homes and private homes) when interacting with their clients about the importance of wearing PPE and having difficulty managing higher-than-usual expectations of the client-provider relationship.

*P20: “When you are doing the orientation you have a mentor, and you have your colleagues doing those meetings with you. I did feel like we're all talking about our stressors. But when the pandemic hit, we had less of those meetings, so it was difficult to connect with colleagues.”*

*P16: “Our team dwindled because no one would see anyone. No one would go into retirement homes. I was the one going into those retirement homes. That was pre-COVID and some of those retirement homes, the clients never wore masks. The clients did not abide [by masking guidelines], so it was scary going in there with [an] outbreak. . .we were not supported like my colleagues in hospitals.”*

From an employer perspective, there were participants who spoke about feeling misunderstood, isolated, and unsupported by their employer and reporting managers. Increased rates of referrals had made some participants feel that best practices and good clinical technique were being less emphasized. They felt that their employer’s focus was on referral management rather than understanding the values and needs of the participants during the pandemic. Many of the participants believed that better communication of expectations of service from their employer to their assigned clients would have been supportive and improved their quality of work life. A few participants identified that their workplace manager was supportive and praised their ability to support them during the pandemic. They felt that they helped them mitigate feelings of occupational and mental stress by under-standing their stresses, explaining evolving changes in public health guidelines, and being available to speak with them. They also referenced participation in a peer support group as improving their quality of work life.

*P5: “I guess that doesn’t sound very positive about my home organization but, they’re toeing the line right. They get these policies and they’re passing them off and so they are doing their job. It just takes away the, the humanity and the caring, it feels like all the stuff about best practice has gone out the window.”*

*P2: “I think the organization has done pretty well, in terms of helping us out. You can’t do everything, right? You still can't control what people do. I think they have done a pretty good job in trying to educate clients. They had a few town halls during the two years and kind of keeping us up to date as well because there is a lot of changes coming up from the government [and] the ministry. Translating some of those orders to how it affects us so that I don't have to do that. That’s helpful.”*

### Theme 3: Personal and workplace coping strategies

Three identified factors influenced the participants' perception of their professional experience during the COVID-19 pandemic: availability and timeliness of workplace support, feelings of professional value, and personal adaptability to stress. These factors were reported to influence their likelihood of maintaining or reducing their home care hours. They were also influential in the development of active coping mechanisms to deal with exacerbations in occupational and mental stress related to working in the pandemic.

### Subtheme 1: Feelings of Professional Value

Despite the challenges presented by the COVID-19 pandemic, the participants understood the importance of providing home care. They acknowledged feeling essential and necessary during the COVID-19 pandemic to ensure the community was serviced medically. They reported that their clients were appreciative and continued to fuel their passion for continuing to work in the home care sector. They received validation from their colleagues and peers who were working in rehabilitation. Many of the participants also acknowledged that the pandemic was a professional growth opportunity. The participants emphasized that it motivated them to be better therapists by further under-standing the importance of home care, especially during a public health crisis. Some participants found ways to be involved in pilot projects, continuing professional education, and seeking more advanced clinical opportunities. Many of the hcRPs with roles outside of home care maintained part-time employment status in home care as they found that home care provides more opportunities to develop and refine their clinical acumen, and that it provides an invaluable service to clients. Those who reported high job importance were found to maintain their hours and engagement in home care during the pandemic.

*P19: “I have stayed because again I really feel like that sense of efficacy that I’m making a difference. I love that I can like see that and I have that relationship with those clients. And I feel it’s also the only job where I’m working with that age group, those babies. There’s not another place to do that. And I’ve done a little bit of private work, the same job but just privately and I always find I am always saying to people, I want them to know first that they can go through home and community care and get the same service for free, right.”*

*P2: “Yeah, I think COVID has raised more questions. Over the last two years, you realize, it's opened the window up a little bit in terms of how valuable home care is. I think that's one really good thing. I'm enjoying kind of the quality of my work, I feel more fulfilled because I feel it's more important especially now more than ever for that to be happening at home whenever possible.”*

### Subtheme 2: Personal Adaptability to Stress

Some participants acknowledged that a rehabilitation professional’s perception and experience of working during the pandemic could be influenced by their self-efficacy. Those who reported experiencing lower occupational and mental stress generally shared a positive disposition about the pandemic, a personal belief in their ability to withstand stress, or an active coping mechanism. The most common coping mechanism implemented was boundary setting or striving to maintain a balance between professional and personal life. Some examples of other active coping mechanisms reported include the practice of spirituality, self-monitoring, physical activity, and emotional support from family and friends. A couple of participants reported that they sought out Employee Assistance Resources for their mental health or consulted with a private therapist. Those who reported experiencing occupational stress and burnout reported that they tried not to reach out for help as much and attributed that tendency to their belief system about asking for interpersonal support. Some participants acknowledged that they chose to work in home care because it gave them the opportunity to be an independent healthcare worker and have a flexible work schedule. This was especially apparent when the participants reported they were a caregiver in their family. There was consistency among the inter-viewed hcRPs that working in home care required a reasonable ability to establish a work-life balance to withstand the effects of work-related stress both prior and during the pandemic.

*P21: “I was actually full-time in the home care setting at that the start of the pandemic in 2020. It was in a way relieving that I was in the homecare setting at that time because there was a lot more flexibility in comparison to facility settings. I could choose to stay home for a week and not see any clients because of safety concerns or not knowing what the state of affairs were.”*

*P6: “It's been different. It started with lots of uncertainties. We didn't know what we were entering into. Then we dealt with it. We were - we were short on PPE. And we dealt with the caseloads, and then we started realizing that no, we're doing a little too much. And we had to step back. And now I think everything is manageable again. So, though it is kind of become an endemic, and we know the symptoms, the clients are a little more aware. I think things are slowly getting back to normal. So, whatever is the new norm is, right? So yeah, overall, it’s been fun. It's fun. Challenging.”*

## Discussion

In this qualitative study, most of the participants reported work-related and mental stress exacerbations related to working during the COVID-19 pandemic. These experiences motivated some participants to reduce their home care hours. Those who reduced their hours in home care had chosen to work in rehabilitation clinics or hospital-based settings as they believed that these offered greater access to workplace support and collegial resources, and reduced job demands compared to their home care role.

COVID-19 presented unique challenges to healthcare professionals. Public health guidelines and social restrictions changed protocols and procedures for managing clients and their caregivers during clinical visits. The COVID-19 pandemic had also exacerbated pre-existing imbalances between job demands and job and/or personal resources in home care rehabilitation professionals. In this pandemic context, hcRPs experienced heightened challenges of managing client expectations, a reduced availability of community service programs to support clients, and greater difficulty accessing assistive devices and medical equipment. Consequently, hcRPs were experiencing job strain related to increased workload, reduced workplace support and resources, and greater provider-client expectations.^35^ These greater job demands in the literature have been associated with health impairments, lower levels of empathy, and decreased physical and psychological functioning linked to work-related stress and burnout.^36-41^

Prior studies of burnout and work-related stress in occupational therapists and other healthcare professionals have stated that healthcare workers have poorly developed coping strategies to withstand prolonged periods of high stress.^15,16,38,39^ In this study, many of the coping strategies were behavioral and therefore, may not be effective during long-term periods of stress as observed in the COVID-19 pandemic. Although some organizational support strategies were recognized such as supportive/ understanding managers and Employee Assistance Programs (EAPS), utilization and accessibility of these resources were not universal amongst the participating hcRPs. Future research should develop a workplace intervention aimed at implementing cognitive coping strategies for home care rehabilitation professionals. The development of such future interventions should recognize diversity in the population, and it’s needs.

Prior studies of healthcare workers during the COVID-19 pandemic have identified elevated burnout rates and reduced mental well-being associated with worries of viral transmission and reduced availability of workplace resources and support.^42,43^ One study reported^44^ that reduced availability of workplace resources and support (e.g., poor communication and insufficient PPE access) was associated with reduced mental wellbeing in healthcare workers related to anxiety about COVID-19 transmission. Global studies of occupational therapists and physiotherapists working during the COVID-19 pandemic reported high rates of personal, work-related, and client-related burnout.^36,37,45^ A study of occupational therapists working during the COVID-19 pandemic^46^ reported higher perceived levels of workplace support and job satisfaction were correlated with lower rates of experiencing burnout. The findings of this study were consistent with global studies on hcRPs working during the COVID-19 pandemic. However, these findings are not unique to the COVID-19 pandemic, as a meta-analysis on burnout in occupational therapists^22^ found that similar variables of engagement, job satisfaction, professional identity, and feeling valued being correlated with burnout.

The job demands-resource (JD-R) model can be used to further characterize our data into a theoretical framework that explains the relationship between job demands and job resources.^21,47^ The primary work stress and burnout factors identified in this study can be classified as job demands (e.g., workload and role strain, lack of colleague collaboration and socialization, workplace environment, fear of transmission of the COVID-19 virus, and client non-adherence to public health guidelines). These job demands can affect these hcRP’s psychological and physical health, leading to increased turnover, decreased work engagement, or risk of burnout.^47,48^

Previous studies have identified a link between psychological functioning and reduced work engagement in healthcare workers.^11,12,49^ The findings in this study suggest that hcRPs reported a decrease in engagement with home and community care through a reduction of clinical hours in this setting – sometimes to leverage the different organizational and collegial resources available in institutional settings. Additionally, some of the identified job demands were organizational in nature and can be affected by work for multiple employers at any given time. Although numerous pandemic-related demands were mediated by the employer (e.g., supplies of personal protective equipment, infection control policies, reduced access to colleagues), many related to healthcare system-wide challenges that are likely to have been common across most employers in the sector. Further investigation of the experiences of hcRPs working for a greater range of employers will help to inform the degree to which organizational policies and resources varied during this time.

The job demands and resource (JD-R) model proposes that these occupational characteristics, defined as workplace demands and resources, are associated with an individual's motivation to work, subsequent performance, and mental and physical wellbeing.^50,51^ According to this model, workplace resources should outweigh workplace demands in each occupational context to promote wellbeing and reduce occupational stress and burnout. In our study, the three factors influencing job resource availability were workplace support, job importance, and self-efficacy. Work in the home care sector involves significant autonomy which, while perceived as positive pre-pandemic, became the primary stressor for these hcRPs who felt that they required greater workplace support than was available during the pandemic. The increased demands of the COVID-19 pandemic added to pre-existing job demands for healthcare organizations and workers were evident in this study. Therefore, applying the JD-R model, the imbalance of heightened job demands, and reduced job resources could ex-plain the experience of burnout and exacerbated job-related stress that led many participants to reduce their clinical hours in home care, reduce work engagement, and subjectively reporting experiencing burnout.^50^

Lastly, it is plausible that personal resources (e.g., feelings of job importance and self-efficacy) could influence the perception of workplace resource availability, work-related stress, and experience of burnout while working in home care.^52,53^ A study^53^ determined that job importance and personal resources mediated the relationship between job resources, engagement, burnout-related exhaustion, and their perception of job resources. They proposed that higher levels of these personal factors may operate on a cognitive basis and be associated with better time management skills, adaptability, and greater capability to manage their work environment. Therefore, we speculate that those who reported greater personal resources in our sample of hcRPs may have the perception of being less affected by work-related stress and/or be more resistant to reduced availability of workplace resources compared to those with lower personal resources.^48,52,53^

In this study, we found that hcRPs who perceived lower workplace support and had lower feelings of job importance had subjectively reported experiencing greater occupational stress and burnout. We speculate that this may be related to work engagement and the hcRPs relationship with six areas of work life^54,55^ including workload, control, community, reward, fairness, and values. Our findings suggest that those with increased experience of occupational stress thereby re-ported higher workloads, lower sense of control, low socialization, and collaboration (community involvement), low reward, and a mismatch between fairness and values between themselves and their employer. Therefore, strategies to improve hcRPs well-being at work, in the context of the pandemic, should target these areas and social job resources.^56^ These areas have been incorporated into three key recommendations for development of hcRP support programs.

### Recommendation 1: Greater opportunities for peer support and mentorship

Peer mentorship and colleague support were embraced as mitigating factors to occupational and mental stress experienced in the pandemic. Self-directed and peer-led hcRP meetings occurring on a weekly or bi-weekly basis to provide mentorship support, case management, and a sense of community within independent hcRPs would be beneficial based on prior research.^57^ Many of the participants acknowledged that their feelings of occupational stress were lessened when they spoke with their colleagues and shared their experiences. These meetings could be supplemented with a peer mentorship program where an experienced and tenured hcRP is matched with an inexperienced hcRP to help them develop effective coping strategies when dealing with work-related stressors, including higher workloads and difficult client interactions. This mentorship would also allow for further experiential training on complex cases in the community that may precipitate work-related stress. This recommendation targets the areas of community, workload, and control.

### Recommendation 2: Recognition and appreciation

Many of the hcRPs in this study felt neglected or underappreciated for their efforts in the community. Providing renewed focus on recognizing individuals for their performance and giving them the opportunity to celebrate success among their colleagues could be beneficial.^58^ Open communication from the employer to these hcRPs to validate their experience and opportunity for reward should be encouraged. In addition to recognizing success, there should be further opportunities to exchange feedback, to improve upon existing best practices and workplace barriers. These recognition opportunities could also be an opportunity to understand the professional values of hcRPs. This recommendation targets the area of reward, values, and fairness.

### Recommendation 3: Development of community resiliency

The perceptions of work-related stress are implicated by personal resources and personal adaptability to stress. Studies have shown that workshops utilizing an adapted group version of mindfulness based cognitive therapy (MBCT) have improved feelings of anxiety, stress, and depression in healthcare workers.^59,60^ A study^61^ found that healthcare workers enrolled in an 8-week MBCT program had improved ability to manage work-related stress, colleague-related stress, and feelings about the quality of their work. Providing opportunities of engagement in workshops to develop resiliency and or build skills through mindfulness-based cognitive therapy could support those who have lower capability to adapt to stress. It can also support a sense of community and improve individual’s ability to self-monitor for signs of occupational and mental stress. This recommendation targets workload, control, and community.

## Study Limitations

There are some noteworthy limitations that may influence our findings. First, the qualitative interviews occurred retrospectively after several waves of COVID-19. Inter-views were also conducted at two separate time points in 2022. This may have influenced reported feelings of occupational and mental stress given that stressors, resource availability, and support systems had changed over time during the COVID-19 pandemic. Second, the sample was recruited via convenience sampling from a single home care rehabilitation employer. Although the interviewer and analysts were all from an external organization, some participants may have under or overreported feelings of occupational and mental stress based on their employment status. Third, the sample was entirely female and primarily occupational therapists in Ontario, Canada, and therefore, may not be representative of hcRPs across Canada. Future research should consider sampling from multiple home care employers in different geographic areas to further investigate perceptions of workplace resources and support among hcRPs to mitigate burnout and stress in the home care sector.

## Conclusion

A reduction of hours within home care rehabilitation results in lasting implications on vulnerable populations, including seniors, disadvantaged, and disabled persons. The findings of this study extend current home care and occupational stress literature through qualitative data analysis of community hcRPs within Ontario, Canada by highlighting proximal contributors to occupational and mental stress. This study also provides insights into some of the reasons that home care rehabilitation providers chose to increase or reduce the hours spent working in home care during the COVID-19 pandemic. With a focus on the effects of COVID-19 on the practice of home care, this study provides a unique perspective on the challenges experienced by hcRPs during an emergent and evolving global public health concern. The exploratory nature of this research works towards providing a framework of factors to examine when creating sustainable and contextual hcRP support programs that aim to benefit the community stakeholders and health care providers. The experiences of hcRPs operating during the COVID-19 pandemic can be used to develop recommendations to further support long-term hcRP occupational wellbeing and increase accessibility of social job resources.
